# Different effects of accelerated development and enhanced growth on oxidative stress and telomere shortening in amphibian larvae

**DOI:** 10.1038/s41598-017-07201-z

**Published:** 2017-08-08

**Authors:** Pablo Burraco, Carmen Díaz-Paniagua, Ivan Gomez-Mestre

**Affiliations:** Ecology, Evolution, and Development Group, Department of Wetland Ecology, Doñana Biological Station, CSIC, E-41092 Seville, Spain

## Abstract

Organisms react to environmental changes through plastic responses that often involve physiological alterations with the potential to modify life-history traits and fitness. Environmentally induced shifts in growth and development in species with complex life cycles determine the timing of transitions between subsequent life stages, as well as body condition at transformation, which greatly determine survival at later stages. Here we show that spadefoot toad larvae surviving pond drying and predators experienced marked alterations in growth and development, and in their fat reserves, oxidative stress, and relative telomere length. Tadpoles accelerated development but reduced growth and consumed more fat reserves when facing pond drying. However, oxidative stress was buffered by increased antioxidant enzyme activity, and telomeres remained unchanged. Predators caused opposite effects: they reduced larval density, hence relaxing competition and allowing faster development and enhanced growth of survivors. Tadpoles surviving predators metamorphosed bigger and had larger fat bodies, increasing their short-term survival odds, but showed signs of oxidative stress and had shorter telomeres. Developmental acceleration and enhanced growth thus seemed to have different physiological consequences: reduced fat bodies and body size compromise short-term survival, but are reversible in the long run, whereas telomere shortening is non-reversible and could reduce long-term survival.

## Introduction

Selection in heterogeneous environments often results in the evolution of adaptive phenotypic plasticity^1, 2^. However, phenotypic alteration is often costly, costs referred to as ‘production costs’^3^. Known production costs involve physiological alterations such as immune state suppression or increased metabolism, which result in changes in life-history traits and ultimately affect fitness^4^. Understanding the physiological effects of plastic alterations of the phenotype will help us assess their short-term and long-term consequences^4, 5^.

In vertebrates, stress responses to environmental challenges are orchestrated via activation of neuroendocrine pathways, being the hypothalamic-pituitary-adrenal (HPA) axis the most studied. HPA-axis activation induces the mobilization of metabolites^6^ but may result in immunological imbalances^7^ that produce profound alterations in developmental and growth rates^8, 9^. Such environmentally induced alterations of growth and development are particularly relevant in species with complex life cycles^10^ because the timing of transitions between life stages and body condition at transformation greatly determine survival at later stages^11^. Accelerating or slowing development and growth have marked consequences across most taxa, ranging from changes in protein turnover within tissues^12^, to allometric changes in body shape and degree of ossification^13^, to variation in fat storage^14^. From a molecular perspective, enhanced developmental and growth rates may result in two major physiological alterations with extensive consequences for fitness: telomere shortening and oxidative stress^15, 16^.

Telomeres are non-coding repetitive terminal regions of the chromosomes specialized in chromosome protection from deterioration, or from fusion with other chromosomes^17^. Telomere sequences are restored via reverse transcriptase telomerase that adds telomeric repeats (TTAGGG in vertebrates) to 3′ overhang. Critical telomere shortness stops cell division and initiates a state of replicative senescence leading to programmed cell death^18^. Telomere shortening is thought of as an internal clock that could potentially be used for estimating chronological age in the wild, although telomere length can vary at different rates over different ontogenetic stages across species. Patterns of telomere length variation over time can be rather complex, as for example in the edible dormouse (*Glis glis*), a hibernating rodent, where telomeres elongate from ages 6 to 9 years^19^, much like in humans telomeres elongate during the development of stem cells, B cells or some tumor cells^20–22^. Therefore, spontaneous ontogenetic shifts from telomere elongation phases to shortening phases, combined with environmentally induced alterations of the rate of telomere shortening loosen the link between biological and chronological ageing^15, 16^. Interestingly, the fact that telomere shortening is susceptible to physiological adjustments due to the environmental conditions experienced provides the means to evaluate the relative costs and trade-offs of phenotypic responses to environmental challenges, especially in early ontogenetic stages^16, 23^.

Developmental acceleration and enhanced growth have been shown to cause telomere shortening as observed *in vitro* in rat pancreatic islets^24^ probably as a consequence of multiple cell divisions, a phenomenon referred to as the ‘end replication problem’^25^, and of oxidative damage accumulated over the cellular lifespan^26, 27^. Accelerated development and enhanced growth, as well as acute episodes of environmental stress, produce excess reactive oxygen substances (ROS) that can result in severe oxidative stress and damage cell structures such as telomere sequences^5^. Both reduced telomere length and increased oxidative stress seem to play a key role in ageing and are good predictors of individual lifespan^5, 28^ although further evidences are still needed^29^.

Most systems studied so far in the context of the interplay of oxidative stress and telomere shortening as a consequence of environmentally induced phenotypic responses have focused on taxa in which growth and development tended to be rather correlated, such as mammals (mainly humans^26^) and birds^30^. However, amphibian larvae have been much less studied in this context despite being an ideal system for evaluating the consequences of developmental and growth plasticity separately. The development of most amphibian species include abrupt ontogenetic switch points in which timing is usually highly plastic since larvae readily modify their activity, morphology, differentiation rate, and growth rate in response to environmental cues^31, 32^. Two main environmental hazards for amphibian larvae are predators and pond drying^33^. Tadpoles are capable of detecting and responding plastically to both risks. However, responses against pond drying and predators seem to be opposite in many respects. Thus, amphibian larvae accelerate development and decrease growth under pond drying conditions^34^. In contrast, predators induce reduced activity and metabolism of amphibian larvae^35^. In addition, predators directly reduce larval density hence relaxing competition and allowing the surviving larvae to reach metamorphosis faster and at a larger size^36^. Analysing the physiological consequences of changes in growth and development in amphibian larvae is important to understand both short-term and long-term carry-over effects of adaptive plastic responses.

Here we evaluate the effects of altered developmental and growth rates in western spadefoot toad tadpoles (*Pelobates cultripes*) as a consequence of pond drying and presence of freely roaming predators. We examined the physiological consequences of such alterations in development and growth on the surviving larvae of each treatment in terms of their fat body content, oxidative stress, and telomere length after metamorphosis. We expected tadpoles to accelerate development in response to pond drying, but at the expense of metamorphosing at a smaller size and with reduced fat reserves. We also hypothesized that pond drying would involve telomere shortening and oxidative damage as a consequence of the increased metabolic effort required for developmental acceleration. Similarly, we expected reduced larval density due to predation to result in lower competition, hence providing better growing conditions for the surviving larvae. Therefore, we expected individuals surviving predators to have a larger mass at metamorphosis, and more abundant fat reserves. In terms of oxidative stress, high resource availability could entail increased metabolism and ROS production, which would have to be balanced with increased antioxidant enzyme activity. Fast growing individuals would be expected to have undergone a greater number of rounds of cell replication, resulting in shortened telomeres.

## Results

### Survival

During the experiment 324 individuals survived and completed metamorphosis out of the initial 960. Of these, 79% kept color markings (VIE tags, see Methods) when they reached metamorphic climax (Gosner stage 42) and could therefore be assigned to sibship.

**Figure 1:**
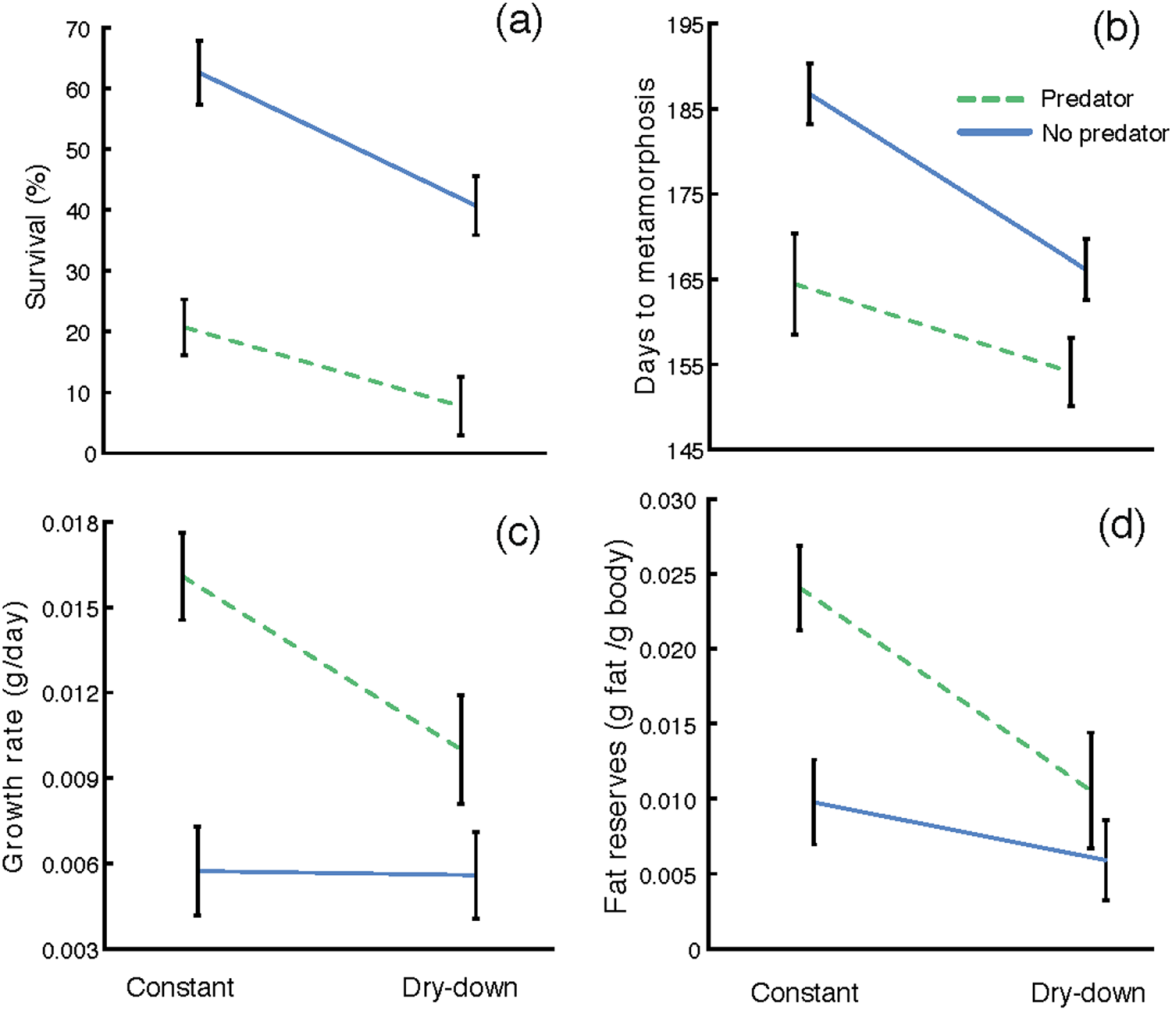
The effect of pond drying and predators on (**a**) survival, (**b**) larval period (days to metamorphosis), (**c**) growth rate, and (**d**) fat reserves in *Pelobates cultripes* metamorphosing from different larval conditions. Data are least square means ± standard error. The water level regime is indicated as ‘Constant’ and ‘Dry-down’ and the lines indicate the presence (P, green dashed line) or absence (NP, blue solid line) of predators.

Pond drying significantly reduced tadpole survival by 41.88% (df = 1, 959; χ^2^ = 12.005, p < 0.001; Fig. 1a), whereas predators reduced it by 72.41% (df = 1, 959; χ^2^ = 30.331, p < 0.001; Fig. 1a). However, we did not find a significant interaction between pond drying and predator presence in tadpole survival (df = 1, 959; χ^2^ = 0.241, p = 0.623; Fig. 1a).

### Time to and size at metamorphosis

Pond drying resulted in shorter developmental period of the surviving larvae, as they reached metamorphosis on average 11.25 days earlier than in constant water (df = 1, 323; χ^2^ = 15.265, p < 0.001; Fig. 1b). This represents an average developmental acceleration of 18.72%. The presence of freely roaming predators also resulted in shorter times to metamorphosis for the surviving larvae (df = 1, 323; χ^2^ = 17.713, p < 0.001; Fig. 1b), showing an average reduction of 14.01 days to metamorphosis with respect to tadpoles in the absence of predators (22.88% shorter larval periods on average). Again, we did not find a significant interaction between both factors in duration of the larval period (df = 1, 323; χ^2^ = 0.990, p = 0.320; Fig. 1b).

Size at metamorphosis of surviving individuals was also greatly affected by both pond drying and predators (Table 1), but in opposite directions. Pond drying caused an average reduction in body mass at metamorphosis of 41.85% (df = 1, 279; χ^2^ = 10.653, p = 0.001), whereas direct predation resulted in 58.71% heavier body mass at metamorphosis of the surviving individuals (df = 1, 279; χ^2^ = 24.451, p < 0.001). The interaction between pond drying and predator presence was not significant for body mass at metamorphosis (df = 1, 279; χ^2^ = 0.0798, p = 0.778). In terms of body length (snout-to-vent length), pond drying resulted in 12.37% shorter toadlets (df = 1, 267; χ^2^ = 6.539, p = 0.011; Table 1), whereas predator presence resulted in 20.97% longer ones on average (df = 1, 267; χ^2^ = 23.755, p < 0.001; Table 1). The interaction between both factors was not significant for body length (df = 1, 267; χ^2^ = 1.102, p = 0.2940). Growth rate of individuals surviving pond drying was reduced by 10.05% on average (df = 1, 279; χ^2^ = 6.009, p = 0.014; Fig. 1c), but increased in larvae surviving predators by 18.49% on average (df = 1, 279; χ^2^ = 28.586, p < 0.001; Fig. 1c). The interaction between both factors was significant (χ^2^ = 2146.5, p < 0.001; Fig. 1c).

**Table 1:** Least square means ± standard errors of measurements obtained in surviving spadefoot toad juveniles (*Pelobates cultripes*) exposed to four environmental conditions in 500-L tanks.

*Life-history traits and fat storage*								
	Survival	Days to met.	Growth rate	SNV	Body mass	Fat storage		
High water - No predator	62.22 ± 5.54	186.74 ± 3.55	0.0062 ± 0.0010	21.35 ± 0.90	1.14 ± 0.16	0.0098 ± 0.0028		
High water - Predator	21.01 ± 4.82	164.46 ± 5.91	0.0153 ± 0.0009	27.20 ± 0.91	2.47 ± 0.15	0.0240 ± 0.0027		
Low water - No predator	40.73 ± 5.14	166.14 ± 3.58	0.0055 ± 0.0009	20.17 ± 0.87	0.89 ± 0.15	0.0059 ± 0.0027		
Low water - Predator	7.86 ± 5.14	154.12 ± 3.98	0.011 ± 0.0014	24.01 ± 1.10	1.65 ± 0.21	0.0105 ± 0.0038		
***Oxidative stress and relative telomere length***								
	**CAT**	**GPx**	**GR**	**SOD**	**MDA**	**GSH**_**t**_	**GSH/GSSG**	**T/S**
High water - No predator	74.54 ± 7.45	4.10 ± 0.84	16.68 ± 1.46	22.80 ± 1.36	16.01 ± 2.03	0.051 ± 0.005	5.03 ± 0.33	1.82 ± 0.11
High water - Predator	49.03 ± 7.24	5.47 ± 0.91	13.56 ± 1.56	21.73 ± 1.39	18.72 ± 2.08	0.066 ± 0.005	4.38 ± 0.33	1.49 ± 0.12
Low water - No predator	68.17 ± 7.31	8.83 ± 0.84	20.57 ± 1.48	26.18 ± 1.39	13.51 ± 2.06	0.062 ± 0.005	4.24 ± 0.33	1.78 ± 0.11
Low water - Predator	68.72 ± 11.99	7.83 ± 1.47	17.99 ± 2.53	24.13 ± 2.47	20.40 ± 3.39	0.063 ± 0.009	3.93 ± 0.63	1.57 ± 0.17

The variables measured were: survival (%), days to metamorphosis (days to reach 46 Gosner stage from the egg), growth rate (g/day of development), snout-to-vent length (SNV; mm), and body mass (g). Fat storage was also measured (g). Moreover, we measured the following physiological parameters: catalase activity (CAT; U/mg total protein), glutathione peroxidase activity (GPx; mU/mg total protein), glutathione reductase activity (GR; mU/mg total protein), superoxide dismutase activity (SOD; U/mg of total protein), malondialdehyde (MDA; nmol/ml), reduced glutathione (GSH; mM), the ratio of reduced to oxidized glutathione (GSH/GSSG), and the relative telomere length (T/S ratio).

### Fat bodies

Fat body content was reduced by an average of 19.79% in juveniles that experienced pond drying during their larval development (df = 1, 108; χ^2^ = 6.166, p = 0.013; Fig. 1d). In turn, fat bodies were on average 18.21% heavier in juveniles surviving predators than in those metamorphosing from predator free tanks (df = 1, 108; χ^2^ = 3.886, p = 0.049; Fig. 1d). The interaction between both factors was not significant (df = 1, 108; χ^2^ = 1.944, p = 0.163; Fig. 1d).

### Oxidative stress and relative telomere length

**Figure 2:**
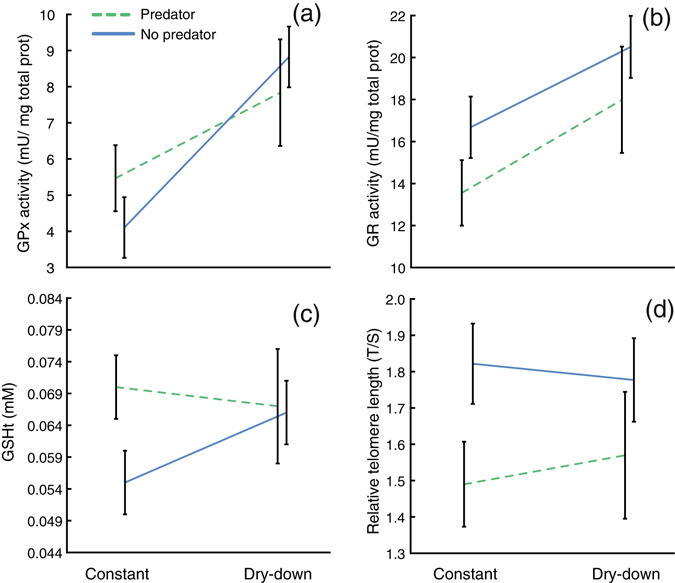
The effect of pond drying and predators on (**a**) glutathione peroxidase (GPx) activity, (**b**) glutathione reductase (GR) activity, (**c**) Total reduced glutathione (GSH_t_) level, and (**d**) relative telomere length (T/S) in surviving juveniles of *Pelobates cultripes*. Data are least square means ± S.E. The water level regime is indicated as ‘Constant’ and ‘Dry-down’ and the lines indicate the presence (P, green dashed line) or absence (NP, blue solid line) of predators.

We found several alterations in the activity of antioxidant enzymes and in oxidative damage in the surviving individuals across experimental factors (Table 1). Neither pond drying nor predation altered CAT activity (df = 1, 104; χ^2^ = 0.302, p = 0.582; df = 1, 104; χ^2^ = 1.707, p = 0.191, respectively). Conversely, individuals surviving pond drying increased their GPx activity by an average of 44.13% (df = 1, 99; χ^2^ = 9.54, p = 0.002; Fig. 2a) whereas individuals surviving predators did not (df = 1, 99; χ^2^ = 0.461, p = 0.497; Fig. 2a). Furthermore, pond drying increased GR activity by 22.20% (df = 1, 104; χ^2^ = 6.647, p = 0.010; Fig. 2b) whereas predators reduced it by 20.91% (df = 1, 104; χ^2^ = 7.630, p = 0.006; Fig. 2b). Finally, SOD activity remained unaltered in individuals surviving either pond drying (df = 1, 104; χ^2^ = 2.297, p = 0.130) or predators (df = 1, 104; χ^2^ = 0.907, p = 0.341). Alterations in the activity of antioxidant enzymes induced by pond drying did not entail changes in lipid peroxidation as indicated by the absence of variation in MDA concentration (df = 1, 103; χ^2^ = 0.084, p = 0.772). Individuals that survived predators showed a non-significant decrease of MDA concentration (df = 1, 103; χ^2^ = 3.504, p = 0.061). Total reduced glutathione (GSH_t_) was 13.47% higher in individuals surviving predators (df = 1, 99; χ^2^ = 4.468, p = 0.034; Fig. 2c) than in those from tanks without predators. However, GSH remained unaltered in individuals surviving pond drying (df = 1, 99; χ^2^ = 2.032, p = 0.154; Fig. 2c). Individuals surviving pond drying experienced a marginally non-significant increase in the GSH/GSSG ratio (df = 1, 99; χ^2^ = 3.499, p = 0.061), whereas those that survived predators did not alter their GSH/GSSG ratio at all (df = 1, 99; χ^2^ = 2.168, p = 0.140; Table 1). We found no significant interactions between both factors in terms of antioxidant responses or oxidative damage (all p > 0.123).

Pond drying did not result in decreased relative telomere length (df = 1, 107; χ^2^ = 0.422, p = 0.516; Fig. 2d). However, individuals surviving predators had lower relative length (15.17% on average; df = 1, 107; χ^2^ = 6.892, p = 0.009; Fig. 2d). We found no significant interaction in relative telomere length between both factors (df = 1, 107; χ^2^ = 0.447, p = 0.504; Fig. 2d).

**Figure 3:**
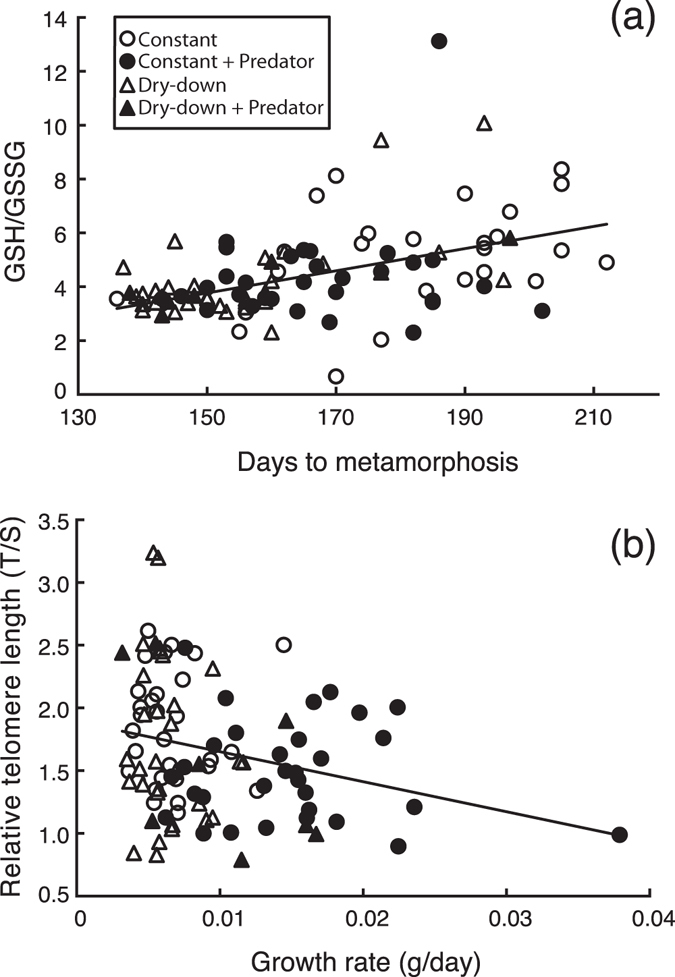
Regressions between (**a**) days to metamorphosis and GSH/GSSG ratio (R^2^ = 0.19, *P* < 0.001) and between (**a**) growth and telomere length (R^2^ = 0.07, *P* = 0.008). Regression lines show the correlation between physiological and life-history traits measured in *Pelobates cultripes* juveniles surviving all four experimental groups combined: constant water level, constant water level plus predator, dry-down, and dry-down plus predators. Cellular oxidative stress normally results in decreased GSH/GSSG ratio, whereas telomere shortening is a reliable indicator of individual/cellular senescence. The GSH/GSSG ratio increased with increased length of the larval period, whereas telomere length was shortened with increased growth rate.

We found a positive correlation between GSH/GSSG ratio and duration of the larval period (Fig. 3a), i.e. tadpoles with faster developmental rates showed lower GSH/GSSG values (R^2^ = 0.19, p < 0.001). This correlation was significant in constant water, low water, and low water plus predator treatments (R^2^ = 0.18, 0.33, and 0.83 respectively, all p < 0.021) but not in the predator treatment (R^2^ = 0.04, p = 0.320). GSH/GSSG ratio and growth rate did not correlate significantly (p = 0.156). On the other hand, relative telomere length correlated negatively with growth rate although the correlation had a low explanatory power (R^2^ = 0.07; p = 0.008: Fig. 3b). We observed a possible outlier in this relationship, with a high leverage on the analysis, showing an elevated growth rate (0.037 g/day). Such a growth rate, however, is within the normal range of growth rates observed for this species, and the individual metamorphosed at 6 g body mass, which is also within the normal size range for metamorphosing individuals in this species. Therefore, we had no biological reasons to discard this data point. Nonetheless, we repeated the analyses without this data point and found that relative telomere length still showed a significant correlation with growth rate, even though the explanatory power was even lower (R^2^ = 0.05; p = 0.024). Relative telomere length did not correlate with duration of the larval period.

## Discussion

In our experimental setup, spadefoot toad larvae experienced environments very similar to realistic conditions found in natural ponds, and consequently the treatments comprising dry-down, predator presence, and especially the combination of both, considerably reduced larval survival. Such differential survival could have had a direct effect on the values observed for the physiological parameters recorded. However, the surviving individuals from the different treatments clearly differed in the level of oxidative stress experienced and in their relative telomere length, indicating that pond drying and reduced competition affected growth and development in rather different ways and with different physiological consequences.

Pond drying reduced larval survival and induced accelerated development at the expense of truncated growth and a smaller size and body mass at metamorphosis. These results are consistent with previous studies analysing plasticity in life history-traits in tadpoles exposed to pond drying^34, 37^. Smaller size at metamorphosis correlates negatively with fitness components in different taxa such as insects^38^, fish^39^, and other amphibians^40^. Developmental acceleration in response to pond drying also caused depletion of fat bodies. Fat bodies are the main lipid storage in amphibians and are determinant of juvenile viability, especially for species that estivate or hibernate after metamorphosis like *P*. *cultripes* ^41^. Moreover, developmental acceleration in response to pond drying induced higher activity of antioxidant enzymes, particularly GPx and GR. Such enhanced antioxidant activity is likely to have been triggered by increased metabolic rate as a consequence of increased corticosterone levels, and the consequent increase in ROS production^37^. However, the redox imbalance seemed to be managed by the antioxidant enzymes as we found no signs of oxidative damage, as indicated by the lack of lipid peroxidation alteration. Moreover, juveniles did not seem to experience intense oxidative stress since GSH_t_, or GSSH levels remained unaltered. Despite the patent poorer condition of juveniles emerging from the experimental pond-drying regime in terms of size, body mass, fat storage, and oxidative stress, their relative telomere length did not vary. This lack of effect on relative telomere length despite incurring in oxidative stress may be due to successful antioxidant activity of enzymes that avoided oxidative damage in the cells.

Amphibian larvae respond to predator cues by reducing their activity and modifying their morphology^42^. Nevertheless, despite these inducible defences, free ranging predators may still have a strong thinning effect on tadpole density, as occurred in this study where the surviving larvae had higher availability of resources and lower intraspecific competition than those not exposed to predators. Consequently, they completed development under relaxed competition and reached metamorphosis quickly, attaining large sizes over the course of a short larval phase. They also metamorphosed with ample fat reserves. This enhanced growth rate was associated with lower antioxidant activity of GR and elevated GSH_t_ levels. GR activity is essential for removal of reactive oxygen species, as it reduces oxidized glutathione (GSSG), which causes oxidative damage in the cells. Previous studies have described decreased activity of antioxidant enzymes^5, 43^, as for example those from tadpoles exposed to herbicide, who decreased their CAT, SOD or GR activities^44^, or cold-stressed rats which decreased CAT and GPx activities^45^. Reduced antioxidant activity compared to benign control conditions could be caused by enzyme inactivation due to excess lipid peroxides and ROS production^46^. In the same individuals, we also observed elevated GSH_t_ levels, one of the most important scavengers of ROS. The maintenance of GSH_t_ levels is crucial in preventing oxidative stress because GSH acts as a chain-breaker of free radical reactions and also because it is the substrate for GPx^47^. For example, increased GSH_t_ has been related to high rates of cellular proliferation in human hepatocellular carcinoma^48^, and is postulated as a mechanism that modulates cell growth by interacting with ROS preventing damage to proteins or DNA and by participating in DNA repair^48^. Observed increments in GSH_t_ levels associated with elevated growth and developmental rates in our experiment are thus congruent with a scenario of oxidative stress, as suggested by the inactivation of antioxidant enzymes, likely due to high ROS production. Tadpoles that survived predators grew quickly and accumulated large fat reserves. The accumulation of fats is linked to increased oxidative stress as well as to elevated GSH_t_ levels, which participates in the improvement of redox imbalance^49^. Moreover, the rate of GSH_t_ synthesis depends on cysteine availability that is obtained directly from the food^50^, which may explain elevated GSH_t_ levels in survivors against predators due to their higher resource availability. However, we only detected a non-significant increase of lipid peroxidation in individuals surviving predators. This may be due to the low specificity of the thiobarbituric acid reactive substances (TBARS) assay, as in this test TBA reacts with a variety of compounds other than malondialdehyde (MDA) like sugars or amino acids, likely interfering its measurement^51^.

Telomere sequences are rich in guanine content and highly sensitive to oxidative stress, which causes single-strand breaks that entail deficient repair of telomeric sequences^5, 52^. It has often been suggested that a trade-off between ROS production and lifespan exists, since oxidative stress is one of the main mechanisms explaining ageing at the cellular level^53, 54^. In addition to unbalanced ROS, telomere shortening seems to be associated with increased growth rate^55–57^. This is probably a consequence of biochemical and physiological alterations at the cellular level, as well as of increased cell replication during enhanced growth^25, 58^.

Nevertheless, most analyses on oxidative stress and telomere shortening have been conducted on birds or mammals. Development and growth are normally tightly correlated in those groups and therefore it is difficult to disentangle whether oxidative stress and telomere shortening are primarily related to enhanced developmental rate, increased growth rate, or both. In contrast, amphibian larvae can largely decouple differentiation rate and growth rate depending on the environmental conditions^13^. Thus, amphibian larvae can typically grow for extended periods of time without advancing in developmental stage, or accelerate development and trigger an early metamorphosis at the expense of truncating growth if conditions worsen. Here we found evidence for increased ROS production, likely linked to developmental acceleration in individuals exposed to pond drying, as indicated by increased activity of antioxidant enzymes. However, individuals undergoing developmental acceleration showed no sign of either oxidative damage or alterations in relative telomere length. In contrast, opposite effects were found in response to predators since enhanced growth rate in surviving juveniles against predators likely favoured increased levels of GSH_t_ and decreased relative telomere length. Our results also suggest a negative correlation between the rate of cell replication during enhanced growth in early larval stages and telomere shortening. Instead, increased metabolic activity as a consequence of accelerated development alone did not result in altered telomere length, although it positively correlated with GSH/GSSG ratio.

In sum, pond drying reduced larval survival and induced accelerated development in spadefoot toad tadpoles, which showed oxidative stress, reduced size at metamorphosis and diminished fat reserves, all of which is known to reduce short-term odds of survival to first reproduction in amphibians^59^. Conversely, predation greatly reduced intraspecific competition, resulting in fast growing tadpoles that metamorphosed large and with ample fat reserves. However, these individuals showed telomere shortening and showed also some signs of oxidative damage, which suggests that despite their greater odds of survival in the short- and mid-term, they may face reduced lifespan. As further studies on oxidative stress and relative telomere length in species with complex life cycles accumulate, we will be able to confirm this pattern where short-term survival is determined by post-metamorphic traits whereas lifespan is linked to the physiological consequences of developmental responses to environmental conditions during early ontogenetic stages.

## Material and Methods

### Bioethics and Animal Care

All experimental procedures in our study were evaluated and approved and euthanasia of juvenile toads were conducted at Estación Biológica de Doñana, CISC, following protocol ‘12_53-Gomez’ approved by the Institutional Animal Care and Use Committee (IACUC) at Estación Biológica de Doñana. All experiments were performed in accordance with relevant guidelines and regulations at the national and European levels.

### Field sampling

In March 2014, we collected between 60–70 eggs from each of ten *Pelobates cultripes* clutches from two different locations in Southern Spain: five clutches from Sierra Norte Natural Park (Seville province) and five from Doñana National Park (Huelva province). While our aim was never to compare across sites, we included in the study clutches from two different areas to ensure that we had substantial variation among families. *Pelobates cultripes* larvae are the biggest amphibian larvae in the Iberian Peninsula, have a long larval period (typically 4–6 months), and are commonly exposed to risk of predation and pond drying. However, *P*. *cultripes* can plastically modify their rates of growth and development^14^ by altering corticosterone and thyroid hormone concentrations, and upregulating expression of hormone receptors^37, 60^.

Individuals from each clutch (family) were raised from eggs for two months prior to the beginning of the experiment. During this period, larvae from each family were raised outdoors separately in 500 L mesocosms (100 cm in height and 120 cm in the upper diameter) filled with 400 L of well water and fed *ad libitum* with natural aquatic plants (*Ranunculus peltatus*, *Myriophyllum alterniflorum*, and *Callitriche obtusangula*), supplemented with rabbit chow. To avoid crowding effects during this initial period we randomly put only 30 tadpoles from each family in each of two tanks. We also collected aquatic beetle larvae (*Dytiscus circumflexus*) in natural ponds and kept them until the onset of the experiment. *Dytiscus* larvae are common and very effective predators of amphibian larvae in the study area.

### Experimental setup

The experiment was conducted in similar tanks as the ones used to initially raise tadpoles. We prepared the tanks for the experiment by adding 130 kg of sand (20-cm deep layer) to each tank plus 5 kg of dry sediment from the basin of several temporary ponds in Doñana National Park to provide a substrate for macrophytes to root in^61^. We then filled each tank with 400 L of well water and allowed macrophytes, zooplankton, and phytoplankton to grow naturally. Tanks were covered with fiberglass window screen to avoid insect colonization. Two months after egg hatching we introduced the tadpoles in the experimental tanks to initiate the experiment. Tadpoles were between Gosner stages 27 and 30^62^ at the onset of the experiment. We crossed two levels of the factor water regime (constant high water level or simulated pond drying) with the presence or absence of predators in a 2 × 2 experimental design. In each tank we put 4 tadpoles from each family for a total of 40 tadpoles per tank. This larval density is well within the range commonly observed in the field^61^. Each treatment was replicated 6 times for a total of 24 tanks. In order to keep track of the different families once mixed in each tank and to be able to control for possible family effects on physiological measurements, we marked all tadpoles with Visible Implant Elastomer (VIE) tags (Northwest Marine Technology, Inc.). VIE tags were introduced subcutaneously in the dorsal part of the body with a 29-G insulin syringe (BD Micro-Fine Insuline U-100 0.5 ml). We used 5 different colours (yellow, red, pink, green and blue) of VIE tags that were put in the back or the front of the head, therefore using 10 different combinations of VIE tags to distinguish the 10 families used in the experiment. VIE tags and positions (anterior or posterior position along the body) were randomized among families and tanks to avoid possible sensory biases from predators towards specific VIE tags that could have resulted in biased predation risk with respect to family identity. That way, different families were tagged with randomly assigned colours and positions in each experimental tank. Marked tadpoles were monitored during 24 hours in 10-L tanks after VIE tags were introduced. All tadpoles survived 24-hours after VIE tag implantation.

One predator was introduced in each tank randomly assigned to the predator presence treatments. Predators (dytiscid water beetles) were first placed in cages for two days using lidded 1-L plastic buckets left afloat and with small holes drilled at the bottom. Caged predators hence provided water borne chemical cues and allowed tadpoles to build up behavioural anti-predatory responses, which are activated immediately upon exposure to predator cues. On the third day, we released the *Dytiscus* larvae inside each tank, allowing them to prey on tadpoles for only 14 days to avoid prey depletion. After that period, predators were caged again in the same floating cages until all surviving tadpoles had metamorphosed. Tanks assigned to predator absence contained empty floating cages. In the tanks assigned to simulated pond drying we removed 30 L of water twice weekly until only a column 10 cm high remained (approximately 50 days after the onset of the experiment), thereafter keeping the volume constant until the end of the experiment. In those tanks assigned to constant water level we added water weekly to keep a constant water level of 80 cm high (400 L).

Tanks were surveyed every other day to retrieve metamorphosing individuals (i.e. individuals at forelimb emergence, Gosner stage 42). Metamorphs were individually maintained in 500 mL lidded cups with 2 mm of pond water at the bottom until they completed tail resorption (Gosner stage 46). Then, they were blotted dry and weighed to the nearest 0.0001 g (intra-individual CV% = 0.15; Intraclass correlation coefficient (ICC) = 0.99). We photographed all juveniles and used the images to estimate snout-to-vent length (SVL) using Image J 1.46r (NIH, USA). Finally, juveniles were euthanized by immersion in a lethal concentration of MS-222, then snap frozen in liquid nitrogen and stored at −80 °C. For fat storage, antioxidant enzymes activity and relative telomere length determination we randomly selected the same subset of 33 marked juveniles from each treatment, except for the combined pond drying plus predator treatment for which we only retrieved 11 marked juveniles recovered at the end of the experiment. Therefore, oxidative stress and relative telomere length measurements were conducted in the same individuals, although in different parts of the body. For oxidative stress assays we used whole eviscerated individuals because the amount of tissue required for assaying the different enzymes was elevated. Instead, for relative telomere length assays we used only a small portion of the leg muscle.

### Fat bodies

Randomly selected juveniles were thawed and dissected for determination of weight of fat bodies on an analytical balance with resolution to the nearest 0.0001 g (CP324S, Sartorius).

### Oxidative stress

We determined the activity of four antioxidant enzymes: catalase (CAT), superoxide dismutase (SOD), glutathione peroxidase (GPx), and glutathione reductase (GR). We also measured malondialdehyde (MDA) formed during lipid peroxidation, and the oxidized and total reduced glutathione (GSSG and GSH_t_, respectively), as indicators of oxidative cell damage. Tadpoles were eviscerated to avoid possible interferences of the intestinal content and were immersed in a buffered solution to inhibit proteolysis (100 mM Tris-HCl with 0.1 mM EDTA, 0.1% triton X-100, pH 7.8 and 0.1 mM PMSF^44^) and were homogenized at 35,000 rpm with a Miccra homogenizer (Miccra D-1). We used a proportion of 1 g of homogenized tadpole in 4 mL of homogenization buffer (1:4, w:v). We centrifuged the homogenates at 20817 g for 30 min at 4 °C and aliquoted supernatants into several 0.6 mL tubes and stored at −80 °C. We determined total protein content and we quantified CAT, SOD, GPx, GR, and MDA according to standard methods (for details and references see Appendix S1) as well as GSH_t_ levels and the ratio GSH/GSSG (see Appendix S1).

### Relative telomere length

We extracted genomic DNA for relative telomere length determination from leg muscle, using a commercial kit for genomic DNA isolation (QIAGEN DNAeasy Blood&Tissue Kit). Genomic DNA was stored at −20 °C until assayed. We measured relative telomere length from a single tissue (leg muscle) to avoid possible differences in this measurement among cell types^63^.

Relative telomere length assays were performed using quantitative PCR (qPCR)^15^. This is an efficient and high-throughput method for measuring telomeric repeats in vertebrates despite potential confounding amplification in some species of interstitial TTAGGG sequences located outside of telomere regions^64^. We used glyceraldehyde-3-phosphate dehydrogenase (GAPDH) as a control, whose forward and reverse primers sequences were 5-AACCAGCCAAGTACGATGACAT-3′ (GAPDH-F) and 5′-CCATCAGCAGCAGCCTTCA-3′ (GAPDH-R), respectively. Forward and reverse of the target gene was 5′CGGTTTGTTTGGGTTTGGGTTTGGGTTTGGGTTTGGGTT-3′ (Tel1b) and 5′-GGCTTGCCTTACCCTTACCCTTACCCTTACCCTTACCCT-3′ (Tel2b), respectively. We performed qPCR for GAPDH and telomere genes on two separated plates using 20 ng of genomic DNA from each sample. The combined set of primers (Tel1b/Tel2b and GAPDH-F/GAPDH-R) was used at a concentration of 900 nM/900 nM in a final volume of 25 μL containing 12.5 μL of Brilliant SYBR Green QPCR Master Mix (Stratagene; Bize *et al*.^15^). PCR cycles for telomere fragment amplification consisted in 10 min at 95 °C followed by 30 cycles of 1 min at 56 °C and 1 min at 95 °C whereas for GAPDH fragment were 10 min at 95 °C followed by 40 cycles of 1 min at 60 °C and 1 min at 95 °C. All qPCRs were carried out on LightCycler 480 (Roche). We tested the efficiency of each qPCR plate performing a standard curve by serially diluting a pool of samples from the different treatments (160, 40, 10, 2.5 and 0.66 ng of DNA per well) in triplicate. We calculated the Cycle threshold (C_t_) value of the reference sample for each plate. We run all experimental samples in duplicate and we used the mean values to calculate the relative T/S ratios, where T is the telomere repeat copy number and S is the single control gene (GAPDH) copy number, by applying the following formula^65^:

$${\rm{T}}{\rm{/}}{\rm{S}}\,{\rm{ratio}}=[{({{\rm{E}}}_{{\rm{telomere}}})}_{{\rm{t}}}^{{\rm{\Delta }}{\rm{C}}\,{\rm{telomere}}\,({\rm{control}}-{\rm{sample}})}]/[{({{\rm{E}}}_{{\rm{GAPDH}}})}_{{\rm{t}}}^{{\rm{\Delta }}{\rm{C}}\,{\rm{GAPDH}}\,({\rm{control}}-{\rm{sample}})}]$$

where E_telomere_ is the real-time PCR efficiency of telomere portion; E_GAPDH_ is the real-time PCR efficiency of the GAPDH portion; ΔC_t_ telomere is the C_t_ deviation of control – sample of the telomere portion; ΔC_t_ GADPH is the C_t_ deviation of control – sample of reference of GADPH (gene of reference) portion. We also estimated the specificity of the melting curve to check for possible primer dimers or secondary amplifications, and we found no indication of undesirable amplifications. The repeatability was 95% for the GAPDH assay (i.e. 5% error rate) and 94% for the telomere assay. The average among-plates efficiency was 2.07 ± 0.052 and 1.87 ± 0.014 for GAPDH and telomere plates, respectively. The average within-plate coefficient of variation was 1.09% and 0.97% for GADPH and telomere assays, respectively. The among-plate coefficient of variation was 7.8% for GAPDH and 10.8% for telomere plates.

### Statistical analyses

All statistical tests were conducted in R (R Development Core Team 2014, version 3.0.2). We observed the distribution of residuals and then tested for normality with a Kolgomorov-Smirnov test (lillie.test in package “nortest”, version 1.0–2). We also tested for homoscedasticity of the data using a Barlett’s test (bartlett.test function in “car” package, version 2.0–22). We fitted linear and generalized mixed models to include both fixed and random effects. We run “lmer” (for parametric data) and “glmer” (for non-parametric data) functions using the package “lme4” (version 1.1–7). The variables *tank* and *family* were introduced in the models as random factors. We also tested the significance of the variable *location* in all models but it was non-significant and we excluded it from the models. In all models, we used likelihood ratio tests to determine the significance of each factor. Normally distributed data (body length, days to metamorphosis, GR, GSSG, GSH_t_, and telomere data) were modelled with a Gaussian error distribution. Non-normally distributed data (body mass, growth, fat bodies, CAT, GPx, SOD, and MDA) were modelled with a Gamma distribution except for the analysis of survival, where we used a binomial distribution. We tested the effect of treatments on growth as log(body mass) – log(larval period) and relative fat body content as log(fat body mass) – log(body mass). Relative telomere length and GR values were log-transformed to fit parametric assumptions. Intraclass correlation coefficients were calculated using “ICCest” function (“ICC” package; version 2.3.0). Full statistical results can be found in the Appendix S2.
